# Comparison of Graphene and Carbon Nanotube Saturable Absorbers for Wavelength and Pulse Duration Tunability

**DOI:** 10.1038/s41598-019-53686-1

**Published:** 2019-11-21

**Authors:** Syed Asad Hussain

**Affiliations:** Department of Engineering Science, University of Oxford, Parks Road, Oxford, OX1 3PJ United Kingdom

**Keywords:** Mode-locked lasers, Fibre lasers, Ultrafast lasers

## Abstract

Graphene (Gr) and Carbon nanotube (CNT) saturable absorbers (SAs) are considered as broadband absorbers and have been used in various studies in the past to get a broad wavelength and pulse duration tunable laser. However, the literature lacks a comparative study on these SAs where one will continue to give longer pulses than the other. In this paper we have compared these two SAs and provided a guideline on how to design them for tunable operation. The parameters that have been compared in this research can be used for other broad gain materials and SAs.

## Introduction

Ultrashort lasers can be used in various applications such as a spectroscopy^1^, laser machining^2^, microscopy^3^, surgery^4^, material processing^5^ etc. This is due to their pulse duration which makes the interaction with the material very short and peak powers that are in watt to kilo-watt range are possible, making them useful in comparison to continuous wave (CW) lasers. SAs have been used in various laser geometries, notability solid state and fibre systems to provide self-amplitude modulation inside the cavity to produce ultrashort laser pulses^6^. Until now, various materials have been used as SAs in lasers, for example, semiconductors^6^, Gr^7^, CNTs^8^, MoS_2_^9^, WS_2_^10^ and other similar materials^11–22^. Among these SAs, Gr and CNTs have achieved significant attention as they can be used in applications where wide-band tunability of laser wavelength and pulse duration is required^23,24^. Owing to wide band emission of an Erbium gain fibre, it is possible to achieve tunability by using these SAs in this laser system. Both Gr and CNTs were tested in this laser system^7,25^, however, a comparison of these two that could be applied to other gain systems has not been presented in the past. In this study, we have presented a comparison of these two and provided various situations where their performance will be limited.

## Results and Discussion

### Continuous wave laser

**Figure 1:**
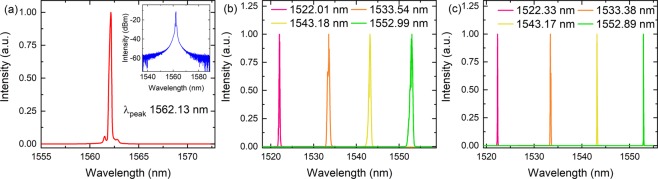
(**a**) Continuous wave spectrum obtained when no filter and SA was used. The inset shows the same spectrum but on a logarithmic scale. By using the same configuration, we can tune the spectrum by using a tunable filter. (**b**) Spectrum when 4.97 nm is used whereas (**c**) was obtained when 1.14 nm was used. In this experiment we have only investigated 1522, 1533, 1543 and 1552 nm as examples, but other wavelengths could also be used.

The laser cavity was first tested without the tuning filter and SA. In this case we obtained a CW laser with the maximum power of 11.13 mW when we pumped the laser at 90.10 mW. The peak of the CW laser spectrum was at 1562.13 nm, Fig. 1(a). The inset of Fig. 1(a) shows the same graph on a logarithmic scale. The CW spectrum could be tuned by using the filter. To test this, we used the 4.97 and 1.14 nm filters. Figure 1(b,c) present tunability graphs from 1522 to 1553 nm. Indeed, other wavelengths could be explored between these wavelengths and beyond this range. The latter case will depend upon the available gain and pump power. This has been presented in other reports and not discussed here^7,26^.

### Mode-locked laser without filter

**Figure 2:**
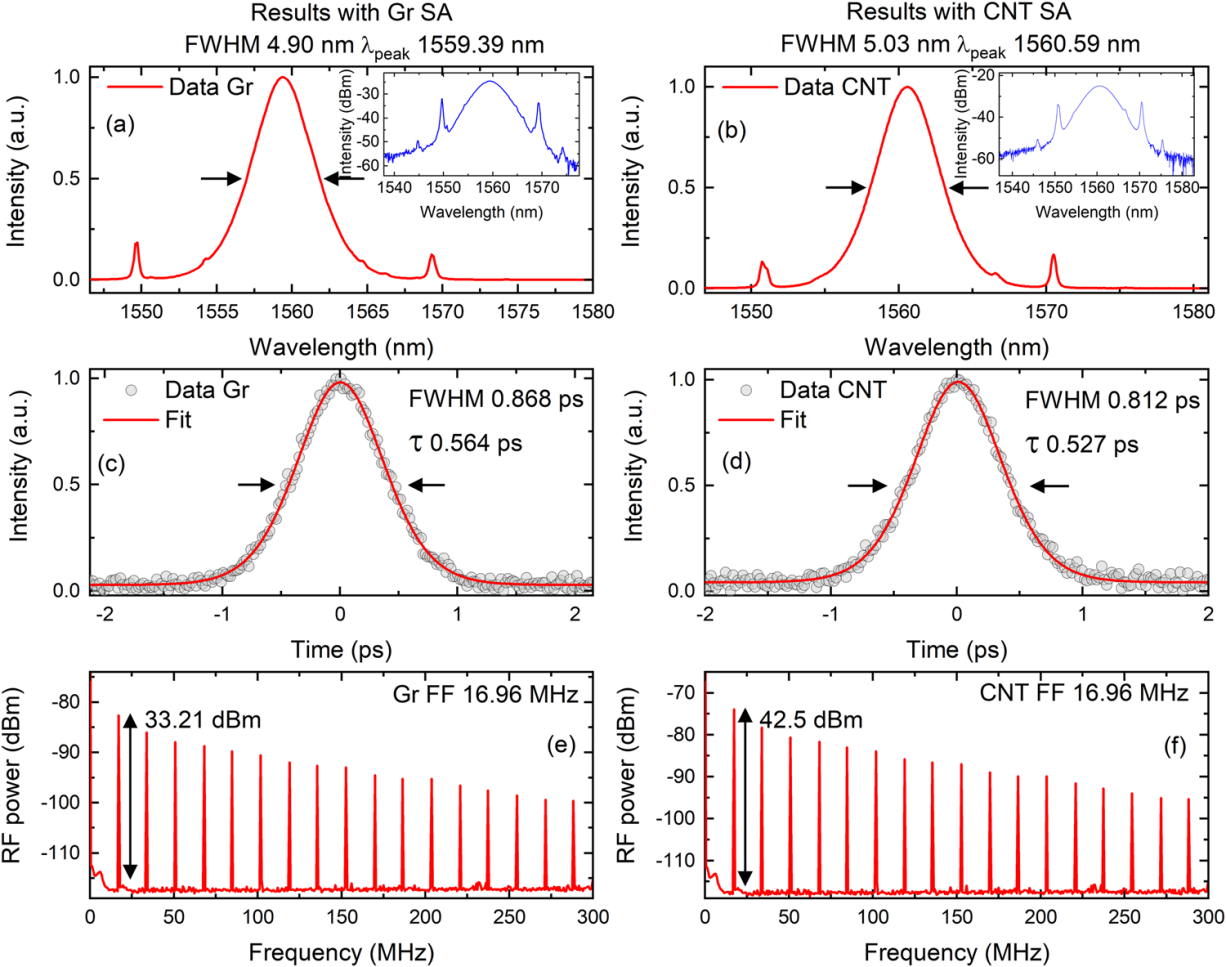
Obtained results for Gr and CNT. The left column represents results for Gr and right for the CNT. Mode-locked spectrums for Gr (**a**) and CNT(**b**). The insets show graphs on a logarithmic scale. Pulse durations for two SAs Gr (**c**) and CNT (**d**). Radio frequency spectrums obtained are also presented for Gr (**e**) and CNT (**f**). The fundamental frequency (FF) for both cases was at 16.96 MHz. All spectrums were obtained without a tunable filter inside the cavity.

We then tested the laser without the filter and first tested Gr. We slowly increased the power and observed two different regions of interest. In the first case we did not tap the optical cavity and mode-locked the cavity at the pump power of 35.52 mW. For this case the obtained average power was 2.39 mW. We later brought the pump power back to zero and increased it again along with gently tapping the cavity. In this case the laser was mode-locked at the pump power of 22.68 mW and provided an output power of 0.97 mW. We tested both cases again by interchanging the order and similar behaviour was observed. The obtained spectrum was independent of the method used and can be obtained by using a commercial spectrometer (Yokogawa, Japan) presented in Fig. 2(a). The obtained spectrum has a peak at 1559.39 nm and FWHM of 4.90 nm. We also observed side bands known as Kelly sidebands. This shows the laser was working in a solitonic regime^27^. To obtain the pulse duration we have used a commercial autocorrelator (APE, Germany). The pulse duration is presented in Fig. 2(b) and was found to be 0.564 ps. Considering a sech^2^ pulse shape, the time-bandwidth product (TBP) was found to be 0.34. Comparing this value with the theoretical value (0.315), this value shows that the obtained pulses were chirped. Having this information at hand, we can now explain the reason of different thresholds for mode-locking. Kärtner *et al*. studied the relaxation time in SAs and defined two types of absorbers - those were fast and slow^28^. If the relaxation is much longer than the pulse duration, the absorber will be termed as slow otherwise fast. To obtain mode-locking in the slow absorber we need to consider the average power inside the cavity as the electrons excited by the photons of the laser pulse will not return during the pulse. Since the mode locking starts from the optical noise inside the laser (Fig. 3 in ref.^29^), it is easy to obtain a self-starting laser for this case. For the case of the fast absorber we need to consider peak power of the pulse as the electrons will be returning from the conduction band within the duration of the pulse. As the pulse duration is 0.564 ps, the pulse duration is almost half of the relaxation time of Gr (~1 ps)^30^. Without tapping we need more optical noise to excite the returning electrons into the conduction band. By tapping we are generating more noise and therefore helping the cavity to excite these electrons. To examine the repetition rate of the laser we have used the information of the radio frequency spectrum. The radiofrequency comb was measured by radio frequency spectrum analyser (Anritsu, Japan) with a span of 300 MHz and RBW of 3 kHz. The fundamental frequency (FF) was found to be 16.96 MHz.

**Figure 3:**
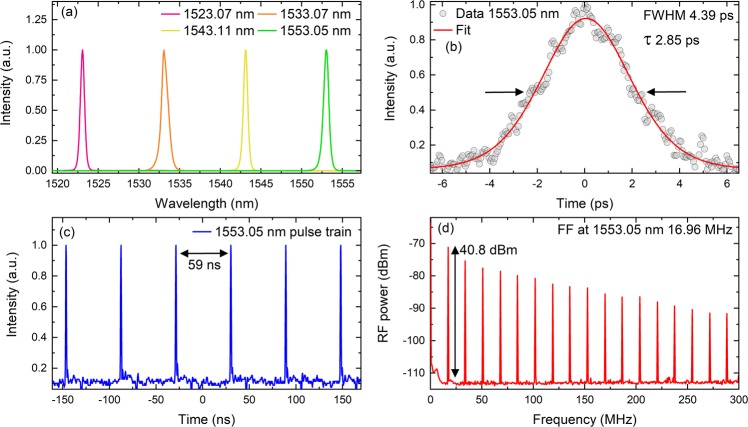
(**a**) Mode-locked spectrums obtained by tuning the filter from 1522 to 1553 nm. (**b**–**d**) pulse duration, pulse train and radio frequency comb for 1553.05 nm. The fundamental frequency (FF) was found to be 16.96 MHz.

We then tested the CNT SA. In this case we tested the tapping phenomena discussed earlier and found no significant difference. This is due to the fact that CNT relaxation is in tens of pico seconds, much greater than Gr^31^. Moreover, from the non-linear transmission results presented in the methods section, we see that the CNT SA started to saturate with very low peak powers. The cavity provided mode-locked pulses at a pump power of 17.09 mW and provided an average power of 0.49 mW. The spectrum obtained in this case was centred at 1560.59 nm with a FWHM of 5.03 nm. We obtained Kelly sidebands in this case as well^27^. The pulse duration was found to be 0.527 ps that resulted in time bandwidth product of 0.33. Since the pulse duration is much smaller than the relaxation time of the CNT, the absorber is acting as a slow absorber and the laser’s inherent noise is enough to generate mode-locked pulses. By comparing it with the theoretical value, the obtained pulses were chirped. Finally, the radio frequency spectrum was found, by using same experimental parameters, to be 16.96 MHz.

### Results with the 4.97 nm filter

After successfully mode-locking the Er cavity without any filter we tested the 4.97 nm filter inside the cavity. By using the CNT SA and without any difficulty we mode-locked the cavity around ~15 mW for all four cases. All four wavelengths that we investigated are shown in Fig. 3(a). The pulse duration, pulse train and radio frequency comb obtained for 1553 nm is presented in Fig. 3(b–d) as an example. The obtained values of all wavelengths are presented in Table 1.

**Table 1:** Experimental results for all the wavelengths obtained with the filter with the CNT SA.

	1523 nm	1533 nm	1543 nm	1553 nm
λ_peak_ (nm)	1523.07	1533.07	1543.11	1553.05
λ_FWHM_ (nm)	0.61	0.95	0.61	0.76
Pulse duration (ps)	4.38	2.39	2.77	2.85
TBP	0.34	0.29	0.21	0.27
Repetition Rate (MHz)	16.96	16.96	16.96	16.96

The pulse durations are in the range of ~3 ps. Except for 1523.07 nm, the TBP is below the theoretical value for sech^2^ pulses. This is due to the spectrums that were not symmetrical for these wavelengths^32^. This could be because of the filter used in the cavity. Comparing these results with the results that were obtained for CNT without a filter, we can easily control the peak wavelength of the laser. Unfortunately, the average powers decreased when we used the filter. Moreover, the Kelly sidebands were not present. This shows that the filter can quench dispersive waves that give rise to these sidebands. A similar effect has been observed when using the filter based on a fibre isolator and polarization controllers^33^.

**Figure 4:**
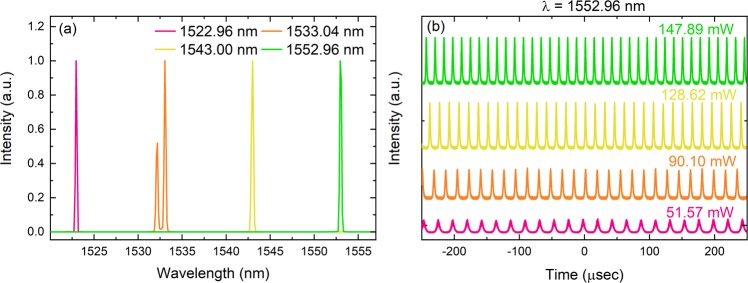
Variation of Q switched spectrums by using a Gr SA. Pulse trains when the pump power was increased from 51.57 to 147.89 mW at 1552.96 nm.

We then tested the cavity for the Gr SA. In comparison to CNT, the Gr SA produced CW a laser beam below 51.57 mW. We increased the pump power and obtained Q switched pulses as shown in Fig. 4(a). The obtained results from 51.57 to 147.89 mW for 1552.96 nm are presented in Fig. 4. These pulses were not stable, and we were not able to reproduce the repetition rate going from a higher power to a lower power. After a few seconds of operation, the laser ultimately switches to CW and occasionally no laser at all. This showed that the sample was slowly burning. The reason why we entered a Q switched regime could be due to high modulation depth, high power density required for saturation, and low intracavity energy which is due to high non-saturable losses. According to the study presented by Hönninger *et al*. the product of the saturation energy of the laser gain medium (E_L_), saturation energy of the absorber (E_A_) and modulation depth (ΔR), should be lower than the intracavity energy (E_C_)^34^. Since the sample was burnt, it was not possible to reach that energy.

Assuming that we would have obtained same pulse duration as CNT SA (~3 ps) and transform limited pulses, the Gr SA would start to act as a fast SA. In this case we cannot consider average values of power and need to consider peak values. In this working regime, the electrons of the SA excited by the laser pulses would continuously return back within the pulse duration^35^ and therefore the peak power needs to be considered in this case, already explained above. Since all laser geometrical parameters are fixed, we need an average intracavity power of 7 mW to achieve a power density of ~530 MW/cm^2^ to obtain the same modulation depth (~0.56 %) that we achieved for CNT SA when we used the same filter. Since the power requirement is high, we must design the cavity accordingly. This is not a requirement for CNT.

Another problem that we mentioned earlier is non-saturable losses. Normally, non-saturable losses increase when we design a high modulation depth sample. This could be avoided by using a sample that has lower modulation depth as this will also decrease non-saturable losses. However, this will increase the pulse duration of the laser^36^ and then it may not be a self-starting laser for low modulation depth^6^. Sabon *et al*. have studied this effect in detail in ref.^36^ and have obtained a range of modulation depths for which laser produce ultrashort laser pulses. Below this range the modulation depth was low and above this range non-saturable losses were high. Therefore, the SA needs clever engineering for the case of a Gr SA. Finally, another effect that has been presented is gradual degradation of the Gr sample with high power density^37^. Although it has been found that for a single layer of Gr this is around 10 GW/cm^2^, this value will change for other samples as they vary in methods by which they were produced. Similar degradation phenomena have been observed for semiconductor SA in which time to damage, depending upon energy density, varies from 4000 seconds to instant damage^35^.

### Results with 1.14 nm filter

**Figure 5:**
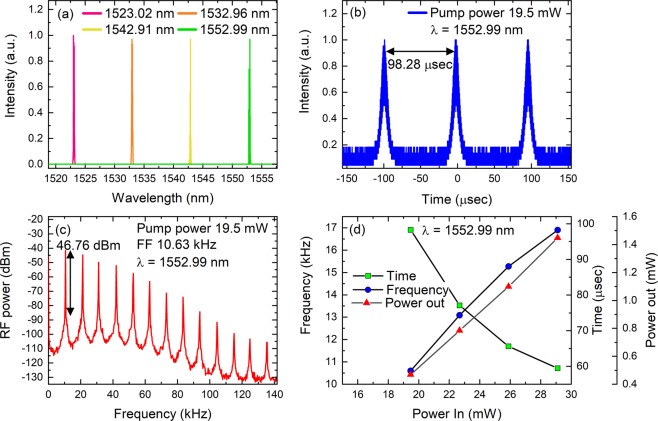
(**a**) Q switched spectrums obtained by using a CNT SA and 1.14 nm filter. (**b**,**c**) pulse train and radio frequency spectrum (RFS) obtained for 1552.99 nm. The fundamental frequency (FF) obtained at 19.6 mW was 10.63 kHz (**d**) Time duration and frequency obtained for various pump powers when the filter’s peak transmission was set at 1552.99 nm.

**Figure 6:**
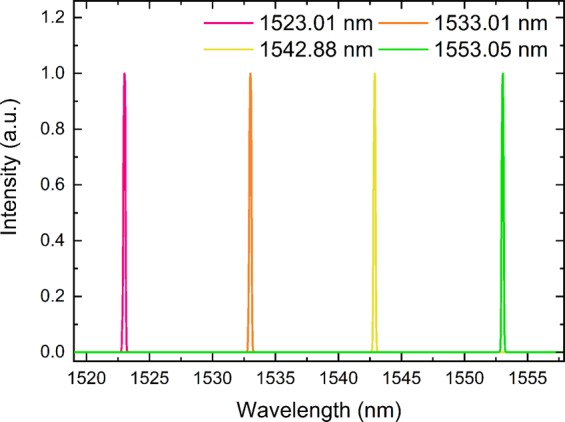
Spectrums obtained when using the Gr SA by using the 1.14 nm filter.

We then switched to 1.14 nm and tested the CNT SA first. In this case we obtained pure Q switched pulses which were in comparison to the Gr results presented for 4.97 nm that were stable and repeatable. Figure 5(a) shows spectrums that were obtained when the tunable filter was tuned. Figure 5(b–d) show results for 1552.99 nm at the pump power of 19.6 mW. Figure 5(d) shows a characteristic increase and decrease of frequency and pulse separation with respect to pump power for Q switched pulses. Considering the same reasoning on fast and slow SAs, the sample was still working in the slow saturable regime as the expected pulse duration (~17 ps) was still lower than the relaxation time of the CNT sample i.e. ~24 ps^31^. In this case the observation of Q switched pulses is purely due to high non-saturable losses as the power inside the cavity is much smaller. It was possible to increase the power and to obtain CW mode-locking. However, as we see for the case of the Gr SA, it was not possible since the SA was burnt. We later introduced the Gr and only observed a CW laser with no Q switched pulses as the power was reduced to a very low level (Fig. 6).

## Conclusion

In this study we have presented a comparison of Gr and CNT SAs by using an Er laser as a test bed. These two SAs have the widest absorption bands and could be used for wavelength and pulse duration tuning. We have found that the CNT SA in comparison to the Gr SA could give much longer pulse duration. We can understand this by using the definition of slow and fast SAs. The relaxation time of Gr is much faster (~1 ps) making it fast SA for any target pulse duration greater than 1 ps. In this case we need to consider peak intracavity power as the electrons excited by the laser pulse will be returning to ground state within the pulse duration of laser pulse. Since the SA will be saturating at peak power which is normally high in comparison to average power, we need to make sure the cavity is able to provide this value. This will mean that we need to make consideration about the peak power otherwise the SA will not provide modulation for mode-locking. Moreover, dependence on peak power may damage or degrade the SA with time which is not good for application point of view. On the other hand, CNT relaxation time is much greater (~20 ps) than Gr. Therefore, for any pulse up to 20 ps it will act as a slow SA and the sample saturation will depend upon average power whose values is lower than peak power. This is due to the fact that electrons will not be returning back into the valance band within pulse duration. This will help us to obtain much longer pulses in CNT SA than Gr SA and will not degrade. For applications where we need wavelength and pulse duration tuning, we should use a SA like CNT with a long enough absorption band and relaxation time. Looking into the available literature we could find studies that have shown wavelength tunability by using these saturable absorbers^38,39^, but only CNT has achieved pulse duration tunability^38^. This study provides the physical reason behind this. Finally, the parameters that have been compared in this research can be used for similar comparative studies for other SAs.

## Methods

### Sample preparation and optical transmission

**Figure 7:**
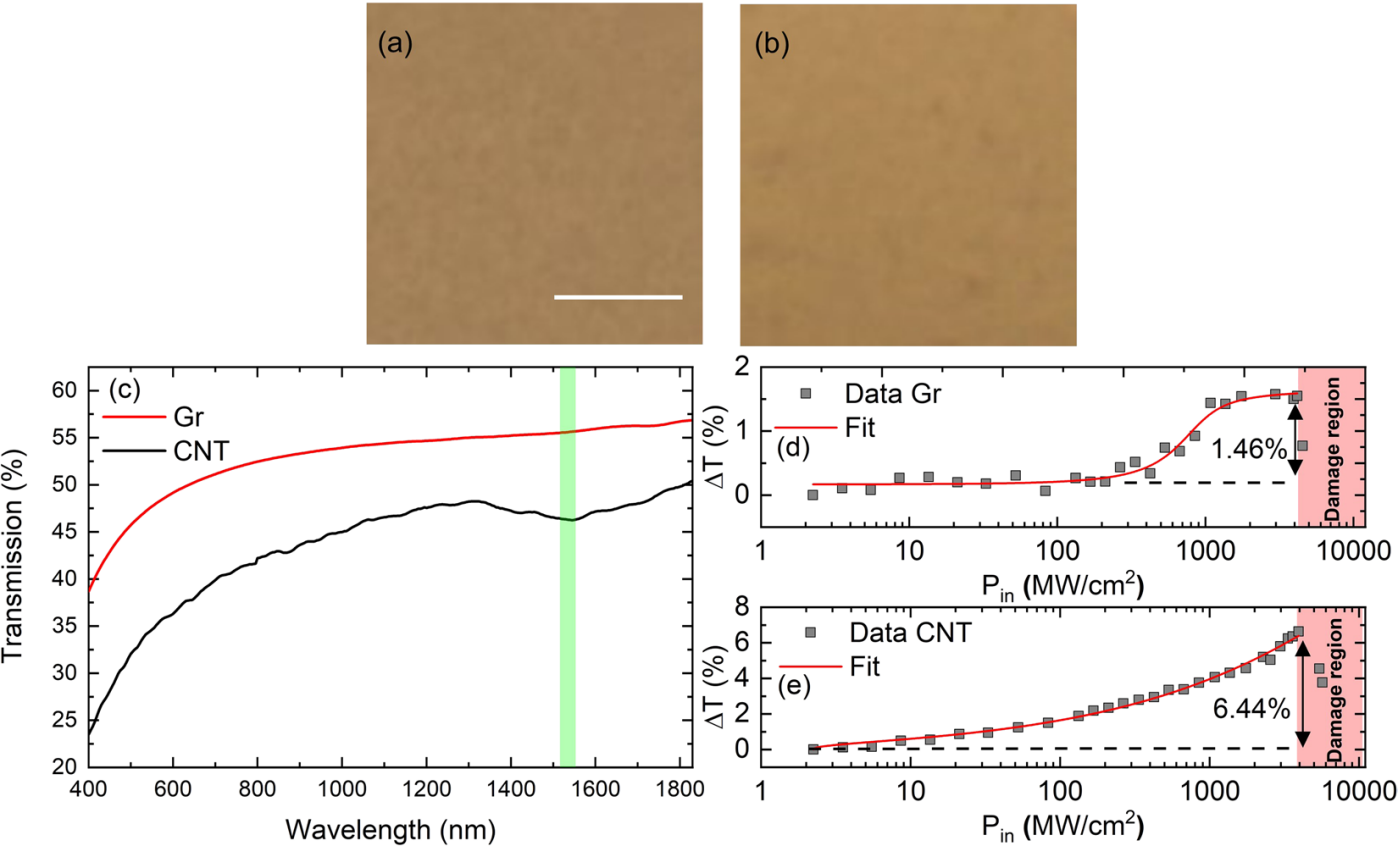
(**a**,**b**) Optical microscope pictures of Gr and CNT samples with a scale bar of 50 μm. (**c**) Transmission of Gr and CNT polymer composite that was obtained from 400 to 1800 nm. Operating region used in the experiment is highlighted with green. The transmission of Gr is almost flat whereas CNT has a big absorption dip around 1550 nm. (**d**,**e**) Non-linear transmission of the samples at 1557 nm by using 392 fs long pulses. The modulation depth is 1.46 and 6.44% for Gr and CNT respectively. We also observed a damage region around 4044 MW/cm^2^ for both samples.

The Gr sample was prepared by ultrasonication of graphite flakes and mixing them with sodiumcarboxymethylcellulose (NaCMC) polymer (with a Gr content of 1 wt%). The resultant mixture was then drop casted and was allowed to dry, which resulted in a ∼30 μm thick Gr-NaCMC composite film^7^. The CNT sample contained single and double walled CNT samples embedded in the NaCMC polymer. It was also obtained by drop casting and was dried in a similar way to achieve similar thickness (∼30 μm) as Gr polymer composite^40^. Figure 7(a,b) shows the pictures of the Gr and CNT samples obtained by using an optical microscope, scale bar 50 μm. Given the core of the optical fibre that is around 8 μm, the samples had no prominent non-uniformities which proves that the process adopted in this paper had provided good quality samples. Linear transmission was measured by using a commercial spectrometer (Agilent, United States) from 400 to 1800 nm, Fig. 7(c). The region that has been used to compare Gr and CNT is represented by a green box. Gr has near constant absorption as reported earlier^41^. Considering 2.3% absorption due to a single layer of Gr^41^, we expect we have around 19 layers in the experimental region. CNT has also decreased absorption in the green highlighted range. The whole absorption dip covers the working wavelengths range very effectively. To measure the non-linear transmission of the samples we used a homemade fibre laser working at 1557 nm providing 392 fs long pulses at a repetition rate of 37 MHz. We cut a small piece of the Gr composite, but big enough to cover the core of the optical fibre (8 μm) and sandwiched the sample between two FC/PC fibre ends. For Gr, the optical power was then increased from 2 to up to 4656 MW/cm^2^. Below 200 MW/cm^2^ we have not observed significant transparency. Beyond this value the sample becomes more transparent and provides saturable absorption. The maximum change in transmission was 1.46%, Fig. 7(d). Beyond ~4044 MW/cm^2^ we damaged the sample and the transmission decreased. Considering 19 layers of Gr the modulation depth (i.e. difference between minimum and maximum transmission, 1.46%) turns out to be very low. This can be due to very high non-saturable losses of the sample^6^. The same procedure was applied for CNT. We observed change in transmission from the very start of increasing power density and reached a maximum change of 6.44%. Beyond ~4044 MW/cm^2^ we discovered the damage region for the CNT sample and the transmission decreased, Fig. 7(e).

### Characterisation by using atomic force microscopy (AFM) and raman characterization

**Figure 8:**
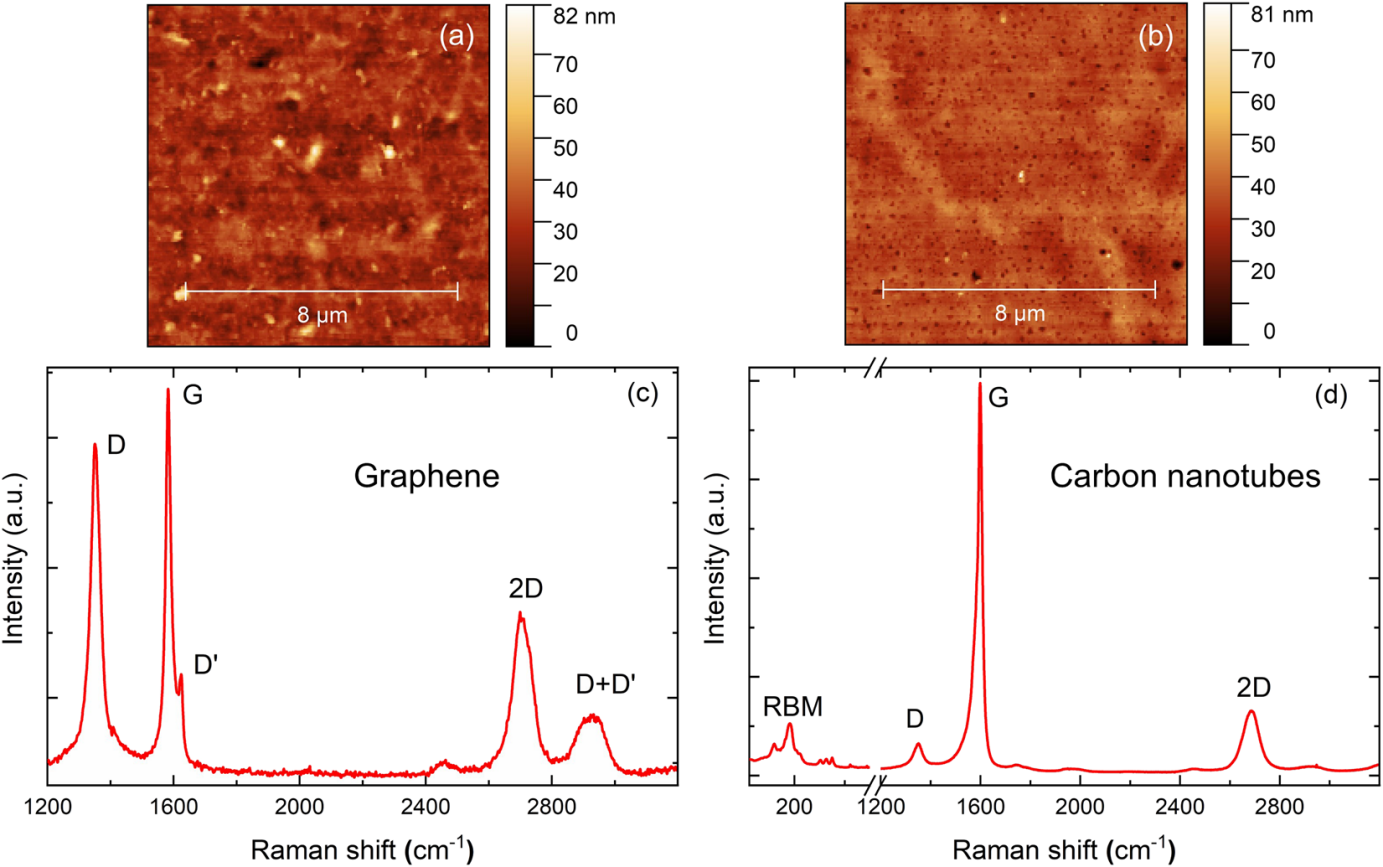
AFM profiles of Gr (**a**) and CNT (**b**) samples. We observed a variation of around 80 nm for both samples. Raman spectrums of Gr (**c**) and CNT (**d**). Spectrum has characteristic G and 2D peaks of sp^2^ carbon systems along with defect peaks D,D′ and D + D′. The Raman spectrum of CNT also has RBM spectrums that can give us information about tube diameters.

To investigate the morphology of the obtained samples in nanometre range, we applied AFM for this purpose. Figure 8(a,b) show the profiles obtained for Gr and CNT samples. Comparing the two samples we observed a variation of around 80 nm. The regions where the profile jumps to approximately 80 nm were not dominant and most of the regions were within 40 nm. Considering the core of the optical fibre (8 μm), the areas that contribute large variation higher than 65 nm are negligible. Raman spectrums were also obtained by using a commercial system (Horiba, Japan). By using an excitation wavelength of 514.5 nm and with a microscope objective of 100 × (Olympus, Japan) we obtained Raman spectrum presented in Fig. 8(c,d) for Gr and CNT. Various peaks were observed. The G band that is present in all sp^2^ carbon bonds system arises due to strain. This peak is present both in Gr and CNTs. 2D like G peak is also present in sp^2^ carbon materials and was found in both samples. D peak is due to defects in carbon schemes. Defects also provide D′ and D + D′. Finally, the Radial Breathing Mode (RBM) position in Fig. 8(d) is linked to tube diameter and therefore only present in the CNT sample^42^.

### Experimental setup

**Figure 9:**
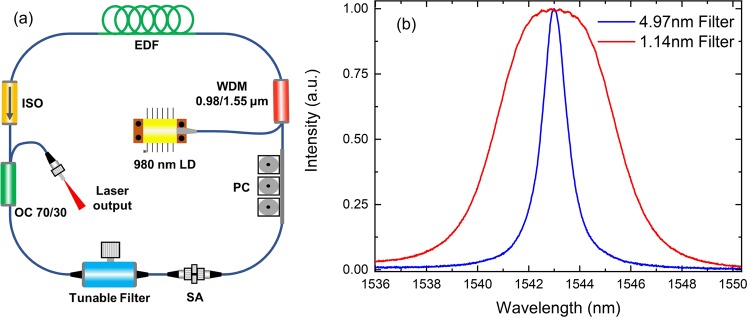
(**a**) Schematic of laser cavity used in the experiment. LD: Laser diode; WDM: Wavelength division multiplexer; EDF: Erbium doped fibre; ISO: Isolator, OC: Optical coupler and PC: Polarization controller. (**b**) Transmission of the tuneable filter. These were exchangeable and removable filters. The FWHM of these filters were 4.97 and 1.14 nm.

The fibre laser used in the experiment is presented in Fig. 9(a). Considering the counter clock wise direction, the laser used an Erbium (Er) doped fibre (EDF) gain medium (Nufern, USA) that was pumped by a 980 nm laser diode. The fibre coupled output of the laser diode was connected to the cavity by using a wavelength division multiplexer (WDM). The isolator (ISO) ensured a unidirectional circulation of the laser pulses. The output of the cavity was extracted by using an optical coupler (OC). We have extracted 70% of the power from the cavity. The rest of the laser power (30%) continued circulating in the cavity. To tune the spectrum of the laser we used a commercially available filter (Koshin Kogaku, Japan) whose bandwidth could be changed by using a changeable filter, whereas the spectrum position of the filter could be modified by manually rotating the screw gauge. Figure 9(b) shows the transmission of the filters which have full width at half maximum (FWHM) of 4.97 and 1.14 nm. The SA was sandwiched between the two FC/PC optical fibres and were joined together by using a fibre connector. To ensure self-starting operation and control of the polarisation we have used a polarising controller (PC). The final length of the cavity was found to be 17.6 m giving a total second-order dispersion of ~0.18 ps^2^.

## Data Availability

Data used in this study can be provided from the author upon receiving a suitable request.
